# Global molecular epidemiology of the incomplete CirA protein related to cefiderocol resistance in *Klebsiella pneumoniae*: a genome-based study

**DOI:** 10.1128/spectrum.01410-24

**Published:** 2025-03-19

**Authors:** Haiyan Long, Yu Feng, Zhiyong Zong

**Affiliations:** 1 Center of Infectious Diseases, West China Hospital, Sichuan University 617661 , Chengdu, Sichuan, China; 2 Division of Infectious Diseases, State Key Laboratory of Biotherapy, Chengdu, China; 3 Center for Pathogen Research, West China Hospital, Sichuan University 34753 , Chengdu, Sichuan, China; University of Pretoria, Pretoria, Gauteng, South Africa

**Keywords:** *Klebsiella pneumoniae*, cefiderocol, antimicrobial resistance, genome analysis

## Abstract

CirA is an iron transporter comprising 657 amino acids in *Klebsiella pneumoniae,* and incomplete CirA alone leads to reduced susceptibility to cefiderocol. We performed a genome-based analysis to study the prevalence of incomplete CirA in *K. pneumoniae* through analyzing all genomes of this species (*n* = 55,517, as of 26 October 2023) available in NCBI. We detected incomplete CirA in 633 (1.27%) genomes with the corresponding strains collected since 1911, across 44 countries on six continents, and mostly (*n* = 563, 88.94%) from humans. Notably, 77 (12.16%) genomes had incomplete CirA in combination with β-lactamases (NDM-1, NDM-5, NDM-7, or KPC-3 plus SHV-11) known to confer cefiderocol resistance. We identified 189 variants of incomplete CirA, including two particularly common ones, a 362-amino-acid remnant due to frameshift by a deletion at *cirA* nucleotide position 1,083 (116/633, 18.33%) and a 562-amino-acid remnant due to premature stop resulting from a mutation at nucleotide position 1,684 (71/633, 11.22%). The 362-amino-acid remnant was mainly found in ST26 (39/116), ST34 (36/116), and ST359 (31/116) strains. The 562-amino-acid remnant was almost exclusive to ST86 (69/71), particularly related to the hypervirulent capsule type K2. Clonal outbreaks (ST26 in USA, ST34 in UK, and ST86 in Vietnam) and cross-border transmission (ST34 in UK and Portugal) were observed. However, this study has limitations, as the analyzed publicly available *K. pneumoniae* assemblies are biased, and only mutations resulting in incomplete CirA were considered. In conclusion, *K. pneumoniae* with incomplete CirA is a global concern, highlighting the urgent need for heightened vigilance and further studies.

**IMPORTANCE:**

Cefiderocol is a critically important antimicrobial agent against multidrug-resistant organisms including carbapenem-resistant *Klebsiella pneumoniae*. We performed a genome-mining study and found incomplete CirA (an iron transporter), which is related to cefiderocol resistance, in a small proportion (1.27%) of publicly available *K. pneumoniae* genomes. However, *K. pneumoniae* strains with incomplete CirA are globally distributed and have been present for over a century, well before the clinical use of cefiderocol. One hundred eighty-nine incomplete CirA variants were identified, suggesting multifactorial causes. Almost all publicly available genomes of ST26, ST34, and ST86 *K. pneumoniae* strains with incomplete CirA have a wide geographic distribution, pointing to the potential existence of particular lineages prone to develop resistance to cefiderocol. Clonal outbreaks and cross-border transmission of strains with incomplete CirA were detected. Incomplete CirA was associated with the hypervirulent K2-ST86 lineage or high-risk multidrug resistance ST16 clone, posing an increased threat or challenge to treatment and infection control.

## INTRODUCTION

Cefiderocol, a novel siderophore cephalosporin, has become an important therapeutic option for treating infections caused by carbapenem-resistant gram-negative pathogens, especially the metallo-β-lactamase-producing organisms (1). However, the rapid emergence of cefiderocol resistance in clinically important gram-negative bacteria has been seen in clinical settings (2–7), mirroring a broader global antimicrobial resistance crisis. Cefiderocol resistance emerges through multiple mechanisms, including mutations in iron transfer systems (notably *cirA* genes), changes in β-lactamase activity (particularly, NDM and PER), alterations in two-component regulatory systems, mutations in penicillin-binding proteins, heteroresistance, efflux pump overexpression, porin reduction, and biofilm-related adaptations (8). Among these pathogens, *Klebsiella pneumoniae* stands out as a particularly concerning threat due to its exceptional ability to acquire diverse resistance mechanisms while maintaining high virulence and transmissibility in healthcare settings. In this pathogen, resistance to cefiderocol has been increasingly reported, often linked to mutations of the *cirA* gene (2, 3, 9, 10). This gene encoding protein CirA, functioning as a specific outer membrane receptor protein, recognizes and binds to cefiderocol-iron complexes, where cefiderocol mimics natural siderophores by chelating Fe^3+^ ions, thereby facilitating the drug’s entry through the bacterial outer membrane via active transport in a process analogous to natural iron acquisition pathways. Notably, these mutations are associated with NDM production, as NDM provides an initial tolerance by marginally hydrolyzing cefiderocol, allowing bacterial survival under drug pressure and subsequently acquiring stable resistance mutations, particularly in the *cirA* gene (11). It has been reported that incomplete CirA due to truncation, interruption, frameshift, or premature stop of *cirA* results in loss of function and can therefore reduce the susceptibility to cefiderocol (2, 3, 6). However, the prevalence and specific patterns of these alterations in clinical isolates of *K. pneumoniae* remain largely unexplored. This study aims to fill this gap by conducting a comprehensive genome-based analysis of publicly available *K. pneumoniae* genomes to uncover the prevalence and characteristics of incomplete CirA, thereby providing a broader understanding of the implications of cefiderocol resistance in this clinically important bacterium.

## MATERIALS AND METHODS

### Retrieving and quality control of *K. pneumoniae* genome assemblies

We retrieved all available *K. pneumoniae* genome assemblies (*n* = 55,517) with their metadata from the National Center for Biotechnology Information (NCBI, https://www.ncbi.nlm.nih.gov/) as of 26 October 2023. Subsequently, we excluded assemblies that were not present in the RefSeq database (*n* = 230), except those associated with large multi-isolate projects. We then used fastANI (12) to verify the species assignation of the assemblies using a ≥95% threshold (12, 13) and excluded those that were not of *K. pneumoniae*. Next, we employed CheckM2 v.1.0.1 (14) for further quality control and excluded assemblies that did not meet the criteria of ≥99.0% completeness and ≤3.13% contamination. The 3.13% threshold represents the upper limit for contamination value data set based on the CheckM2 results, which were not normally distributed; as such, we derived this upper limit using the quartile method. This threshold was chosen to maintain high-quality genome assemblies while accounting for the inherent technical variations in sequencing and assembly processes.

### Identification of sequence types (STs), capsule types, and antimicrobial resistance genes

We assigned the genomes to an ST and a capsule type using mlst v.2.18.0 (https://github.com/tseemann/mlst) and Kleborate v.2.0.6 (15), respectively. We predicted antimicrobial resistance genes in the genomes using AMRFinderPlus v.3.11.26 (16).

### Identifying coding sequences and abnormal length in CirA

We used Prokka v.1.14.6 (17) to predict coding sequences in each query genome. We performed subsequent comparisons of the resulting protein sequences with the CirA amino acid sequence of the cefiderocol-susceptible *K. pneumoniae* reference strain ATCC 13883 (accession no. CP040993.1) using the BLAST+ v.2.15.0 (18). We then retained the highest scoring hits to assess the completeness of the CirA protein encoded by the corresponding query genomes.

### Single nucleotide polymorphism (SNP) calling and phylogenomic tree inference

We included genomes exhibiting discrepancies (*n* = 633) in CirA protein length relative to the reference sequence in further analysis by mapping the contigs against the complete chromosome of strain ATCC 13883 (accession no. CP040993.1) harboring an intact *cirA* gene using Snippy v.4.6.0 (https://github.com/tseemann/snippy) with default settings. A pseudo-alignment was created with the snippy-core script from Snippy, followed by removing non-core sites using SNP-sites v.2.5.1 (19). We used the refined SNP alignment to infer a maximum-likelihood phylogenomic tree using IQ-TREE v.2.26 (20) under the GTR + GAMMA model (the most comprehensive time-reversible model for nucleotide evolution) incorporating ascertainment bias correction and testing with 1,000 bootstraps (a widely accepted standard in phylogenetic analyses that provides robust statistical support for tree topology while maintaining computational efficiency). This SNP-phylogeny pipeline was also applied to the genomes of ST16 (*n* = 21), ST26 (*n* = 70), ST34 (*n* = 215), ST86 (*n* = 265), ST147 (*n* = 8), ST359 (*n* = 51), ST376 (*n* = 3), ST512 (*n* = 3), and ST4843 (*n* = 4) each compared against the reference genome of their respective ST types to allow more accurate estimation (21, 22). Specifically, the comparisons were made to JASUVZ01 for ST16, CP062997.1 for ST26, LR890323.1 for ST34, CP084868.1 for ST86, JALDOQ01 for ST147, CP083768.1 for ST359, ABEYRJ01 for ST376, VNPW01 for ST512, and ABKOGK01 for ST4843. We used a cutoff of ≤21 SNPs to define clonal transmission events, as this cutoff value provides optimal discrimination between closely related isolates at both the country level and, even more strikingly, the hospital level (23).

### Identifying the potential causal reason for discrepancies in CirA length

We annotated variants within the *cirA* coding region and meeting the filtering criteria of a minimum depth of 10 and a fraction of 0.9 with SnpEff v.5.0e (24). Additionally, query genomes, whose variant annotation indicated no alteration in protein length, were further compared with the mobile elements collected in the ISfinder database (25). We specifically searched for insertion sequence (IS) elements within and around the *cirA* gene sequence, as these elements are known to contribute to antimicrobial resistance through gene disruption or by affecting gene expression through insertional inactivation or promoter modification. In our study, an incomplete CirA protein was defined as one with fewer or more amino acid residues than that of the reference strain ATCC 13883, as determined by BLAST results.

## RESULTS

### Variants of incomplete CirA protein in *K. pneumoniae*

We retrieved 55,517 *K. pneumoniae* genome assemblies from NCBI, and after quality control, 49,927 were included for subsequent analysis (Fig. S1). We found that CirA was absent from three genomes and was incomplete in 633 (1.27%, 95% CI: 1.24%–1.30%; 633/49,927). We then calculated the number of amino acids remaining in the altered CirA sequence compared to the complete CirA of the reference strain ATCC 13883 (9). For interrupted sequences, we counted the number of amino acids of the N-terminus. We uncovered that incompleteness-causing modification positions are scattered throughout the entire CirA protein sequence, with the highest frequency occurring at amino acid position 362 due to a frameshift mutation at nucleotide position 1,083 of *cirA* (thereafter referred to as the “362aa-remnant”)*,* followed by position 562 due to a premature stop codon resulting from a mutation at nucleotide position 1,684 (referred to as the “562aa-remnant”) (Table S1).

**Fig 1:**
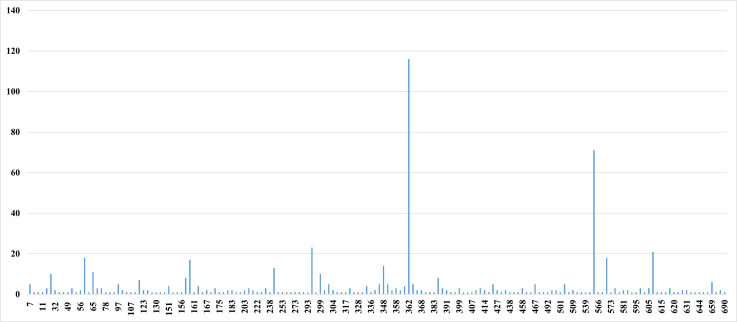
Distribution of incomplete CirA variants according to the remnant amino acid length. The horizontal axis of the bar chart represents the length of the incomplete CirA remnant, and the vertical axis is the quantity. For interrupted sequences, we counted the number of amino acids containing the N-terminus.

The predicted incompleteness of CirA could potentially arise from seven mechanisms identified through sequence analyses, namely mutations causing a frameshift (*n* = 334), mutations resulting in premature stop (*n* = 208), interruption (*n* = 38) (by insertion sequence in 35, prophage in 2, and structure variant in 1), mutations leading to deletion (*n* = 38), truncation (*n* = 8) (by start codon disrupted in four, start lost in two, disrupted by insertion sequence in one, and deletion from start in one), in-frame insertion (*n* = 6) by duplication of nucleotides, and stop lost (*n* = 1) (Table S1). However, it is important to note that these are computational predictions, and experimental validation such as protein gel analyses would be required to confirm the actual presence or absence of the CirA protein. We identified 189 distinct variants of incomplete CirA, among which only four have been reported previously (3, 9, 26), in the 633 genomes (Fig. 1 for the distribution and Table S2 for a list of each variant). Such four variants were due to frameshift by a duplication at nucleotide position 475 or 903 or a nucleotide deletion at position 1,461 or premature stop resulting from a mutation at position 1,281 with all nucleotide positions here and thereafter referring to the *cirA* gene of strain ATCC 13883 (accession no. CP040993.1) (Table S2). Notably, there are four additional variants of incomplete CirA that have been reported in *K. pneumoniae* in the literature (2–4, 10) but were not detected in the 633 genomes. The four reported variants were due to frameshift by a deletion at nucleotide position 703 or 1,300 or a 7 bp duplication spanning positions 761 to 767 or premature stop resulting from a mutation at position 397 of *cirA* (Table S2).

### Global distribution of *K. pneumoniae* with incomplete CirA variants

**Fig 2:**
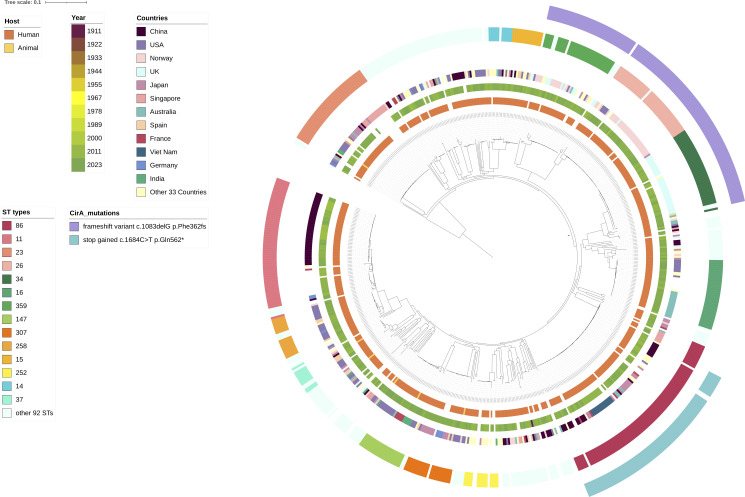
Phylogenetic tree of 633 *K. pneumoniae* strains with incomplete CirA. From the inner circle to the outer circle are hosts, collection years, countries, ST types, and the two most common types of incomplete CirA variants.

We examined the metadata and found that the 633 genomes that encoded incomplete CirA were collected during a long period of 1911–2023, across 44 countries on six continents. Notably, the strain recovered in 1911 stands apart, with a 93-year gap before the collection of the remaining 632 genomes between 2004 and 2023. Among the 633 genomes analyzed, the majority were sourced from China and the USA, each contributing 98 genomes (15.48%), followed by Norway (*n* = 65, 10.27%), UK (*n* = 52, 8.21%), and Japan (*n* = 49, 7.74%), with most (563, 88.94%) of these strains recovered from humans and a small proportion (9, 1.42%) isolated from animals. The 633 strains could be assigned to 106 STs with ST86 (*n* = 80, 12.64%), ST11 (*n* = 61, 9.63%), ST23 (*n* = 42, 6.64%), and ST26 (*n* = 40, 6.32%) being the most common types (Fig. 2; Table S1).

### Co-occurrence of incomplete CirA and β-lactamase genes in *K. pneumoniae*

As abovementioned, incomplete CirA in *K. pneumoniae* alone has been found to reduce susceptibility to cefiderocol but is not adequate to confer resistance (2). Nevertheless, it has been reported in the literature that the co-occurrence of an incomplete CirA with either (i) any of the three NDM-type β-lactamases (NDM-1, NDM-5, or NDM-7), or (ii) a KPC-3/SHV-11 enzyme combination, can confer resistance to cefiderocol (2, 3, 9, 10). We therefore examined the presence of these β-lactamase-encoding genes in the 633 genomes with an incomplete CirA (Fig. S6). We found genes encoding NDM-1 in 36 (5.69%, of 11 STs), NDM-5 in 26 (4.11%, of 8 STs), NDM-7 in 5 (0.79%, of 2 STs), and KPC-3 plus SHV-11 in 10 (1.58%, of 6 STs) genomes, making up 77 (12.16%) of the 633 genomes.

To explore whether there were clonal transmissions, we determined pairwise SNPs for each ST comprising ≥2 strains with the same abovementioned β-lactamase-encoding genes. We detected eight domestic transmission clusters (Table 1). First, there were 15 ST16 strains with an incomplete CirA and NDM-1 comprising 14 from Australia with two to five pairwise SNPs, and one from Thailand, which exhibited 2,384 SNPs with the Australian strains. Second, three ST16 USA strains with incomplete CirA and NDM-5 had 10–12 pairwise SNPs. Third, there were six pairs of strains with ≤21 pairwise SNPs, all of which were recovered from the same country (China, Germany, or USA) (Table 1).

**TABLE 1:** The metadata of combinations able to confer resistance to cefiderocol

Incomplete CirA+	ST	No.	Countries (no.)	Pairwise SNPs	Note^*a*^
NDM-1	ST16	15	Australia (14) Thailand (1)	2–2,384	2–5 SNPs between Australian strains
	ST4843	4	USA (4)	4–238	4 SNPs between two strains
	ST14	2	USA (1) United Arab Emirates (1)	2,496	
	ST307	2	Germany (2)	7	7 SNPs between two strains
	ST359	2	Canada (1) USA (1)	163	
	ST11	1	Poland (1)		
	ST15	1	UK (1)		
	ST37	1	USA (1)		
	ST252	1	Qatar (1)		
	ST313	1	China (1)		
	ST376	1	Germany (1)		
	ST462	1	USA (1)		
	ST1399	1	USA (1)		
	NA	3	China (2) Germany (1)	73–29,341	
NDM-5	ST147	8	USA (5) India (3)	24–2,967	
	ST16	6	USA (6)	10–1,595	10–12 SNPs among three strains
	ST376	3	USA (3)	5–33	5 SNPs between two strains
	ST307	2	USA (2)	9	9 SNPs between two strains
	ST383	2	Egypt (1) USA (1)	256	
	ST5214	2	China (2)	1	1 SNP between two strains
	ST11	1	USA (1)		
	ST313	1	China (1)		
	ST1326	1	China (1)		
NDM-7	ST26	4	USA (4)	38–118	
	ST16	1	USA (1)		
KPC-3 + SHV-11	ST512	3	Spain (2) Swiss (1)	43–309	
	ST258	2	USA (2)	2	2 SNPs between two strains
	ST3603	2	USA (2)	82	
	ST12	1	USA (1)		
	ST37	1	Italy (1)		
	ST462	1	USA (1)		

^*a*^ Only those with ≤21 SNPs are shown.

Considering the possibility that other types of NDM and KPC may also confer resistance to cefiderocol in the presence of an incomplete CirA but have not just been explored by experiments, we examined the presence of NDM and KPC encoding genes in the remaining 556 genomes with an incomplete CirA but no abovementioned β-lactamases. We therefore found 140 additional genomes containing genes encoding KPC, either KPC-2 (*n* = 131) or KPC-3 (but without SHV-11, *n* = 9), but did not detect other genomes containing NDM-encoding genes (Fig. S6). Notably, SHV-encoding genes are intrinsic to *K. pneumoniae* (27). It remains to be determined whether a KPC, in particular KPC-2, in the presence of an SHV (except the known KPC-3 plus SHV-11 combination) and an incomplete CirA can confer resistance to cefiderocol and, if yes, what variants of KPC and SHV in combination would do so.

### Distribution of *K. pneumoniae* with the 362aa-remnant of CirA protein

We characterized *K. pneumoniae* strains harboring a 362aa-remnant of CirA. This genetic feature was predominantly found in three sequence types: ST26, ST34, and ST359. Phylogenetic analysis showed ST26 and ST34 strains clustered on single branches, while ST359 strains were distributed across two branches. Potential clonal transmission was observed in the USA (ST26), UK (ST34), and UK/Norway (ST359).

The 362aa-remnant was detected in 116 genomes from 12 countries, predominantly in three STs: ST26 (*n* = 39, 33.62%; mainly from Norway, 79.49%; others from Russia, South Africa, UK, and USA); ST34 (*n* = 36, 31.03%; mainly from the UK, 91.67%; the other two from Japan and Portugal), and ST359 (*n* = 31, 26.72%; most from Norway, 41.94%) (Table S1). There were 70 ST26, 215 ST34, and 51 ST359 among the 49,947 *K. pneumoniae* genomes. Correspondingly, over half of ST26 (55.71%, 39/70) and ST359 (60.78%, 31/51) strains and 16.74% of ST34 (36/215) ones had 362aa-remnant.

We further inferred respective phylogenomic trees of ST26, ST34, and ST359 strains to examine the distribution of the 362aa-remnant within each ST and determined pairwise SNPs between strains with 362aa-remnant. We found that strains containing 362aa-remnant were clustered on a single branch for each of ST26 (7–277 pairwise SNPs within the ST, Fig. S2 and Dataset S1) and ST34 (1–69 SNPs, Fig. S3 and Dataset S1). In ST26, four USA strains had 7–20 pairwise SNPs, indicating domestic clonal transmission (Dataset S1). For ST34, 17 UK strains shared 1–20 SNPs, while 10 from UK and Portugal had 2–20 SNPs (Dataset S1). In contrast, ST359 strains with the incomplete CirA were clustered in two separate branches comprising 4 (73–183 SNPs) and 27 (5–238 SNPs) strains, respectively (Fig. S4 and Dataset S1). In the larger branch, there are 5 SNPs between the two UK strains and 14 SNPs between the two Norway strains. Strains of the two branches had 213–333 SNPs, and there is another intervening branch comprising strains without 362aa-remnant (Fig. S4 and Dataset S1).

### Distribution of *K. pneumoniae* with the 562aa-remnant of CirA protein

We examined *K. pneumoniae* strains carrying a 562aa-remnant of the CirA protein, finding it predominantly in ST86 strains from East and Southeast Asia. This genetic feature shows potential clonal transmission in Vietnam and is strongly associated with the hypervirulent K2 capsule type.

The 562aa-remnant was found in 71 genomes with the vast majority (*n* = 69, 97.18%) belonging to ST86 (Table S1). These ST86 strains were mainly from China (*n* = 19, 27.54%), Vietnam (*n* = 14, 20.29%), or Japan (*n* = 13, 18.84%) and were also seen in Australia, France, Romania, Russia, Serbia, South Korea, Switzerland, Thailand, and the USA. There were 265 ST86 strains among the 49,947 genomes; correspondingly, 26.04% (69/265) of all ST86 genomes had 562aa-remnant. We also inferred a phylogenomic tree and determined pairwise SNPs for ST86 strains. ST86 strains with the incomplete CirA were clustered on a single branch with 1–514 pairwise SNPs (Fig. S5 and Dataset S1). Notably, seven of these strains, all from Vietnam, showed 5–20 pairwise SNPs, indicating domestic transmission. Notably, it is well known that ST86 is typically associated with the capsule type K2, representing a hypervirulent *K. pneumoniae* lineage (28–30) (Fig. S5 and Dataset S1). We therefore performed capsule typing for ST86 strains and uncovered that 54 strains belonged to K2 and 12 to “unknown (K2)” (Fig. S5), which is a variant of K2 with fragmentation of the K2 locus.

## DISCUSSION

In this study, we investigated the variants of the incomplete CirA protein and their distributions. Our data revealed the global distribution and long-term existence of *K. pneumoniae* with an incomplete CirA even far prior to the FDA’s approval of cefiderocol in 2019. Notably, CirA plays a crucial role in iron acquisition, a function intimately linked to virulence and competitive fitness against other microbes. The observed prevalence of incomplete CirA variants suggests that factors other than cefiderocol exposure drive the emergence of this incomplete form. The diversity of incomplete CirA may reflect bacterial adaptation and evolution in response to changing environmental pressures. Furthermore, previous studies have demonstrated that the presence of NDM can promote the generation of incomplete CirA under cefiderocol exposure. This suggests that other metallo-β-lactamases might influence CirA similarly. Understanding the interactions between resistance mechanisms and iron acquisition could reveal broader patterns in bacterial adaptation and shed light on why incomplete CirA variants persist in clinical settings.

We found that 12.16% of *K. pneumoniae* strains harboring incomplete CirA variants also carried specific β-lactamase genes, such as NDM-1, NDM-5, NDM-7, and the combination of KPC-3 and SHV-11, which can confer resistance to the last-resort antibiotic cefiderocol. This co-occurrence is not an uncommon phenomenon. We identified multiple domestic transmission clusters harboring cefiderocol resistance. Two of them belong to ST16, which is a globally disseminated, multidrug-resistant, high-risk clone of *K. pneumoniae* (31). The existence of these clusters in ST16 highlights the potential for clonal dissemination of cefiderocol-resistant strains across different regions.

Furthermore, we discovered additional genomes containing KPC-2 or KPC-3 (without SHV-11) in combination with incomplete CirA variants. This finding raises questions about the potential for these combinations to contribute to cefiderocol resistance as well. Further investigations are warranted to determine the impact of different KPC and SHV variants on cefiderocol susceptibility when co-occurring with incomplete CirA variants.

We then studied the distribution of the two most common variants of incomplete CirA, 362aa-remnant and 562aa-remnant. The 362aa-remnant was predominantly found in ST26, ST34, and ST359, while the 562aa-remnant was mainly observed in ST86. The distinct distribution patterns of the two variants across different STs suggest that they might have arisen from independent mutational events and subsequently disseminated within their respective clonal populations. Phylogenomic analyses demonstrated that strains harboring incomplete CirA clustered on a single branch in ST26, ST34, and ST86. This indicates a single origin of the incomplete CirA, followed by clonal expansion. Interestingly, in ST359, strains with incomplete CirA were distributed across two separate branches, possibly implying two independent mutational events within this sequence type. The low levels of pairwise SNPs among certain strains indicate evidence of domestic (ST359 in the UK and Norway, and ST86 in Vietnam) and international clonal transmission (ST34 between the UK and Portugal). Notably, the association of the 562aa-remnant with the hypervirulent ST86 lineage is particularly concerning. This lineage, predominantly associated with the K2 capsular type, is already known for co-existence of resistance and enhanced virulence, which may cause severe infection (30, 32). The alarming convergence of this remnant with the ST86 background potentially creates a formidable pathogen combining increased virulence and significantly reduced antimicrobial susceptibility. This evolution not only exacerbates the severity of infections but also complicates treatment strategies, underscoring the urgent need for enhanced surveillance and innovative therapeutic approaches.

Taken together, we uncovered a diverse array of incomplete CirA proteins across strains with a global distribution, spanning over a century, and from varied clonal backgrounds, which may contribute to decreased susceptibility to cefiderocol. Notably, we found many of these strains carried both incomplete CirA and β-lactamases, a combination known to confer resistance to cefiderocol, and some of them were even from regions where this agent has not been approved for clinical use yet, such as China. We also detected the domestic and international clonal spread of strains carrying incomplete CirA, including those belonging to the hypervirulent K2-ST86 lineage or multidrug resistance ST16 high-risk clones. Such associations are clinically relevant, posing an increased threat or challenge to treatment and infection control. Our analysis illustrates a global concern regarding cefiderocol resistance or decreased susceptibility, indicating its prevalence and distribution across multiple countries. This widespread challenge is further complicated by the emergence of various cefiderocol resistance mechanisms, encompassing β-lactamases and mutations in the *cirA* gene. It should be noted that the evolution of *cirA* mutations is linked to the activity of metallo-β-lactamases, such as NDM. Inhibiting the activity of NDM can prevent the emergence of *cirA* mutations, at least *in vitro* (6, 11). This underscores the urgent need for heightened vigilance and rigorous surveillance to monitor the emergence and dissemination of cefiderocol resistance in clinically significant gram-negative bacteria. Deciphering the underlying factors driving CirA truncations is also pivotal for formulating strategies to curb further evolution and prevent the spread of untreatable infections.

We are aware of the limitations of this study. First, we analyzed publicly available *K. pneumoniae* assemblies, while genomes deposited in the NCBI are typically biased and are unlikely to reflect the real prevalence of the carriage of mutations resulting in incomplete CirA in *K. pneumoniae*. Second, we only analyzed mutations resulting in incomplete CirA. However, there are instances of missense mutations that encode single amino acid substitutions in intact CirA and also lead to lost function of CirA (3). Nevertheless, studies of these missense mutations are scarce, and it remains unclear what type of amino acid substitutions occurring in which positions can lead to lost function of CirA in *K. pneumoniae*. We therefore focused on mutations resulting in an incomplete CirA in this study. These limitations may affect our understanding of the true prevalence and impact of incomplete CirA. First, the sampling bias in NCBI-deposited genomes may over- or under-estimate the actual frequency of incomplete CirA in clinical settings, as these deposits often favor isolates with notable resistance patterns or clinical significance. Second, our focus on truncating mutations, while providing clear functional insights, might have overlooked the potential contribution of missense mutations to cefiderocol resistance. To address these limitations, we propose several future research directions: (i) to conduct prospective surveillance to obtain more reliable prevalence data; (ii) to systematically characterize CirA variants through experiments; (iii) to develop molecular diagnostic tools targeting both truncating and functionally significant missense mutations in CirA; and (iv) to conduct longitudinal studies for tracking the evolution of CirA mutations under different conditions and understanding patterns of resistance development. Despite the limitations, we examined about 50,000 publicly available *K. pneumoniae* genomes, allowing the identification of the scale of cefiderocol resistance or reduced susceptibility.

## Data Availability

The genome assemblies analyzed in this study are available in the NCBI database. All supplementary data are freely accessible through Zenodo (DOI: 10.5281/zenodo.14062055).

## SUPPLEMENTAL MATERIAL

Dataset S1 spectrum.01410-24-s0001.xlsx Comparative SNP distance matrices for genomes carrying the 362-amino-acid CirA remnant (ST26/ST34/ST359 lineages) and genomes with the 562-amino-acid CirA remnant (ST86 lineage).

Figure S1 spectrum.01410-24-s0002.docx Flowchart of retrieving and quality control of *K. pneumoniae* genome assemblies.

Figure S2 spectrum.01410-24-s0003.docx Phylogenomic tree of all ST26 strains from the 49,947 *K. pneumoniae* genomes.

Figure S3 spectrum.01410-24-s0004.docx Phylogenomic tree of all ST34 strains from the 49,947 *K. pneumoniae* genomes.

Figure S4 spectrum.01410-24-s0005.docx Phylogenomic tree of all ST359 strains from the 49,947 *K. pneumoniae* genomes.

Figure S5 spectrum.01410-24-s0006.docx Phylogenomic tree of all ST86 strains from the 49,947 *K. pneumoniae genomes*.

Figure S6 spectrum.01410-24-s0007.docx Distribution of β-lactamase genes that may related to decreased cefiderocol resistance in 633 *K. pneumoniae* strains with incomplete CirA.

Table S1 spectrum.01410-24-s0008.xlsx The 633 genomes containing incomplete cirA.

Table S2 spectrum.01410-24-s0009.xlsx Each variant of incomplete CirA.

ASM does not own the copyrights to Supplemental Material that may be linked to, or accessed through, an article. The authors have granted ASM a non-exclusive, world-wide license to publish the Supplemental Material files. Please contact the corresponding author directly for reuse.
