# Supplementary figures and images for: Global molecular epidemiology of the incomplete CirA protein related to cefiderocol resistance in Klebsiella pneumoniae: a genome-based study
Source: Microbiol Spectr. 2025 Mar 19;13(5):e01410-24. doi: 10.1128/spectrum.01410-24 (PMC12054181; doi:10.1128/spectrum.01410-24)

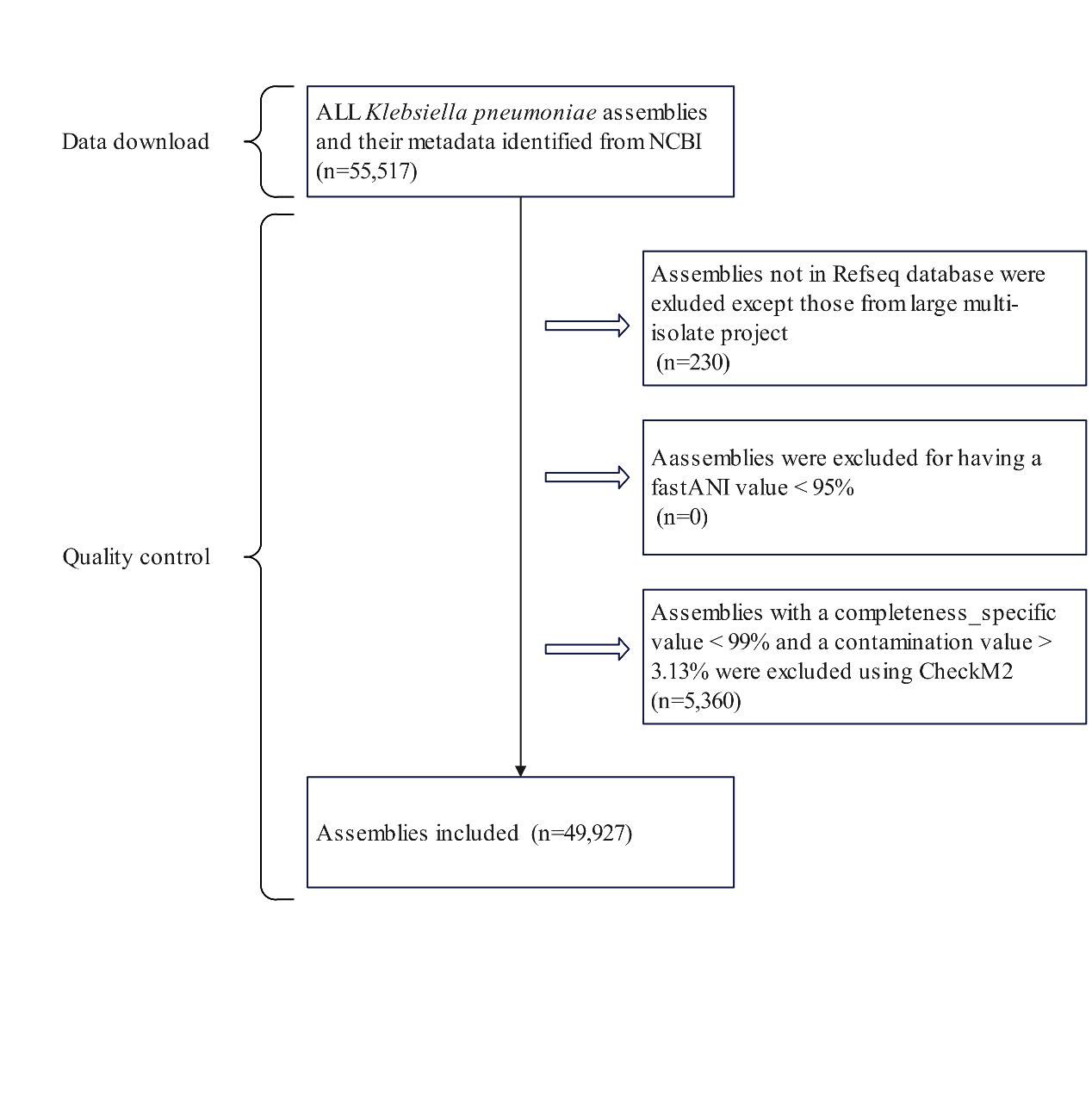


Figure S1. Flowchart of retrieving and quality control of *K. pneumoniae* genome assemblies

Supplement: Figure S1 — Flowchart of retrieving and quality control of K. pneumoniae genome assemblies. [file spectrum.01410-24-s0002.docx]
