# Supplementary material for: Global molecular epidemiology of the incomplete CirA protein related to cefiderocol resistance in Klebsiella pneumoniae: a genome-based study
Source: Microbiol Spectr. 2025 Mar 19;13(5):e01410-24. doi: 10.1128/spectrum.01410-24 (PMC12054181; doi:10.1128/spectrum.01410-24)
Supplement: Figure S3 — Phylogenomic tree of all ST34 strains from the 49,947 K. pneumoniae genomes. [file spectrum.01410-24-s0004.docx]

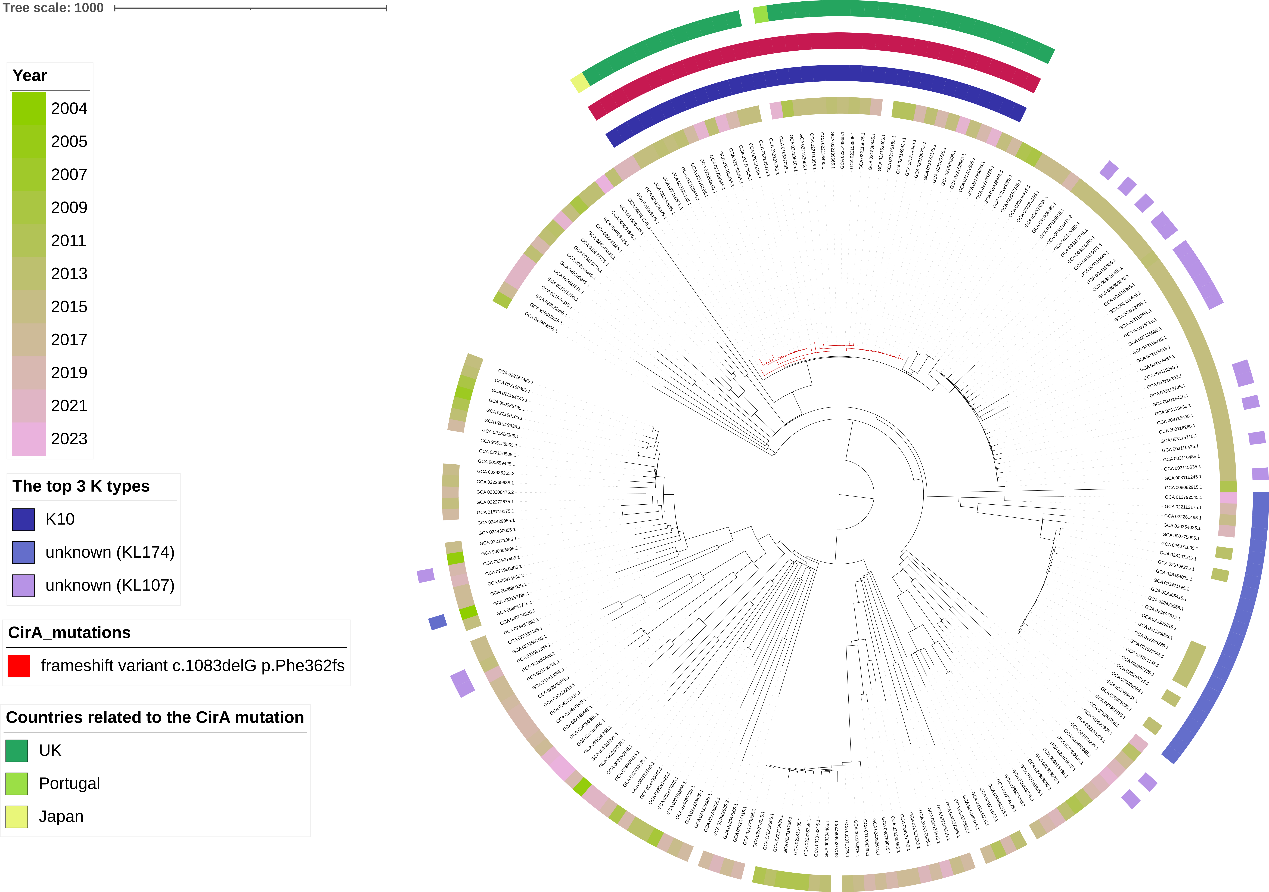


Fig S3. Phylogenomic tree of all ST34 strains from the 49,947 *K. pneumoniae* genomes. The annotation information of this tree from the inner circle to the outer circle are collection years, the top three K types, CirA frameshift mutation caused by a deletion at nucleotide position 1083 and countries related to this variant.
