# Supplementary material for: Global molecular epidemiology of the incomplete CirA protein related to cefiderocol resistance in Klebsiella pneumoniae: a genome-based study
Source: Microbiol Spectr. 2025 Mar 19;13(5):e01410-24. doi: 10.1128/spectrum.01410-24 (PMC12054181; doi:10.1128/spectrum.01410-24)
Supplement: Figure S4 — Phylogenomic tree of all ST359 strains from the 49,947 K. pneumoniae genomes. [file spectrum.01410-24-s0005.docx]

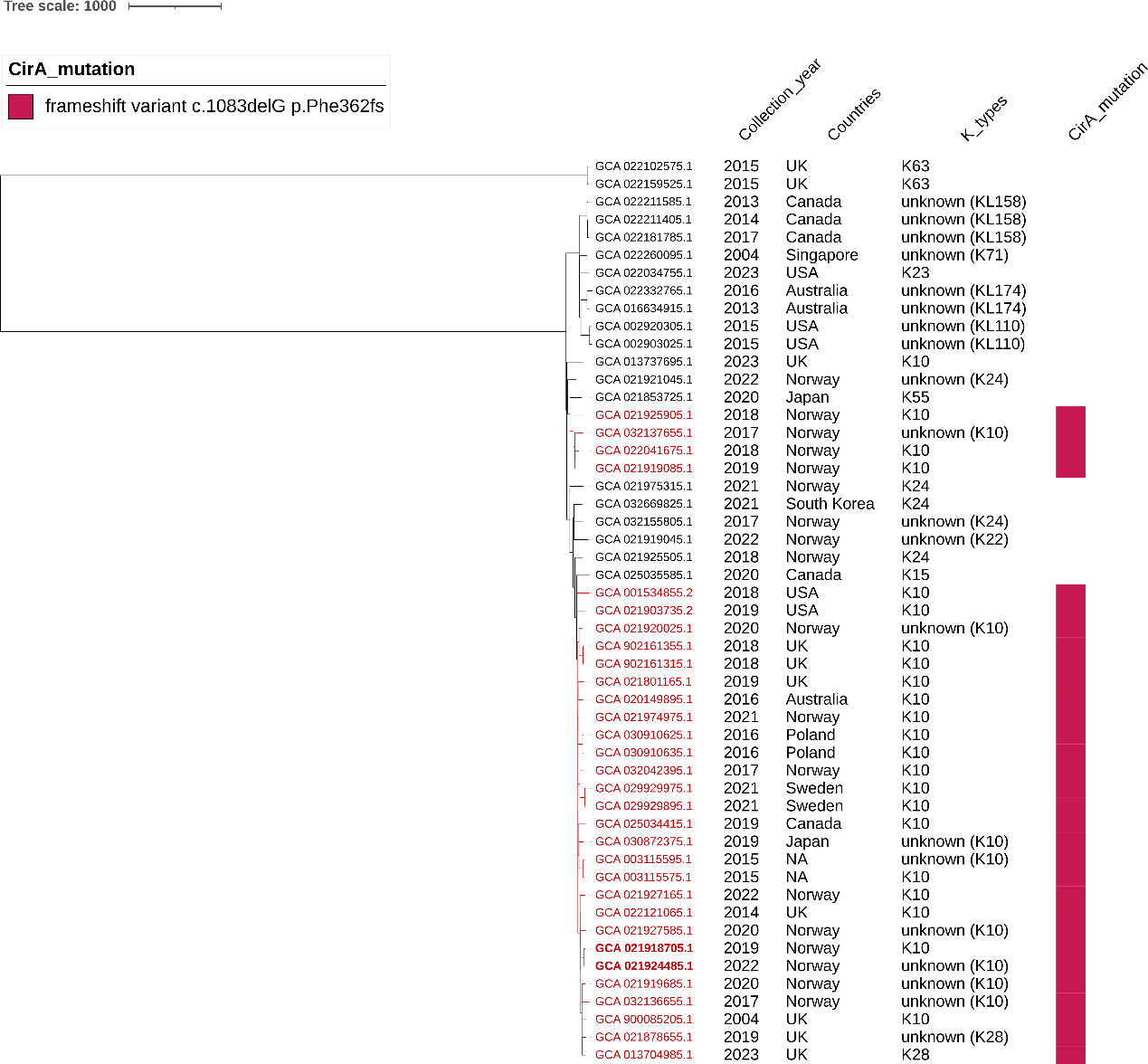


Fig S4. Phylogenomic tree of all ST359 strains from the 49,947 *K. pneumoniae* genomes. The annotation information of this tree from left to right are collection years, countries, K_types and CirA frameshift mutation caused by a deletion at nucleotide position 1083.
