# Supplementary material for: Global molecular epidemiology of the incomplete CirA protein related to cefiderocol resistance in Klebsiella pneumoniae: a genome-based study
Source: Microbiol Spectr. 2025 Mar 19;13(5):e01410-24. doi: 10.1128/spectrum.01410-24 (PMC12054181; doi:10.1128/spectrum.01410-24)
Supplement: Figure S6 — Distribution of β-lactamase genes that may related to decreased cefiderocol resistance in 633 K. pneumoniae strains with incomplete CirA. [file spectrum.01410-24-s0007.docx]

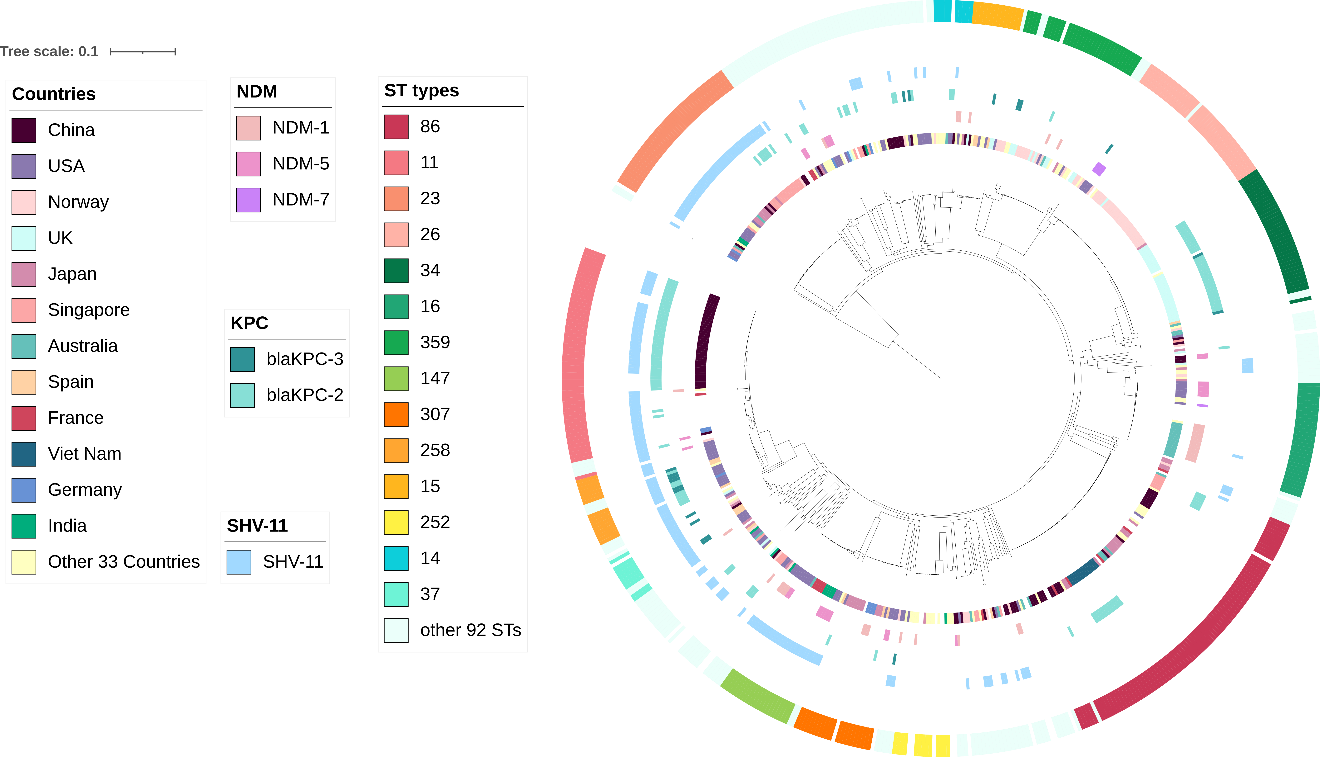


Fig S6. Distribution of β-lactamase genes that may related to decreased cefiderocol resistance in 633 *Klebsiella pneumoniae* strains with incomplete CirA. This phylogenetic tree, from the inner ring to the outer ring, consists of countries, NDM, KPC, SHV-11, and ST types, respectively.
